# Supplementary material for: Stephanoascus ciferrii Complex: The Current State of Infections and Drug Resistance in Humans
Source: J Fungi (Basel). 2024 Apr 18;10(4):294. doi: 10.3390/jof10040294 (PMC11050938; doi:10.3390/jof10040294)
Supplement: Supplementary file 1 [file jof-10-00294-s001.zip › jof-2838128-supplementary.pdf]

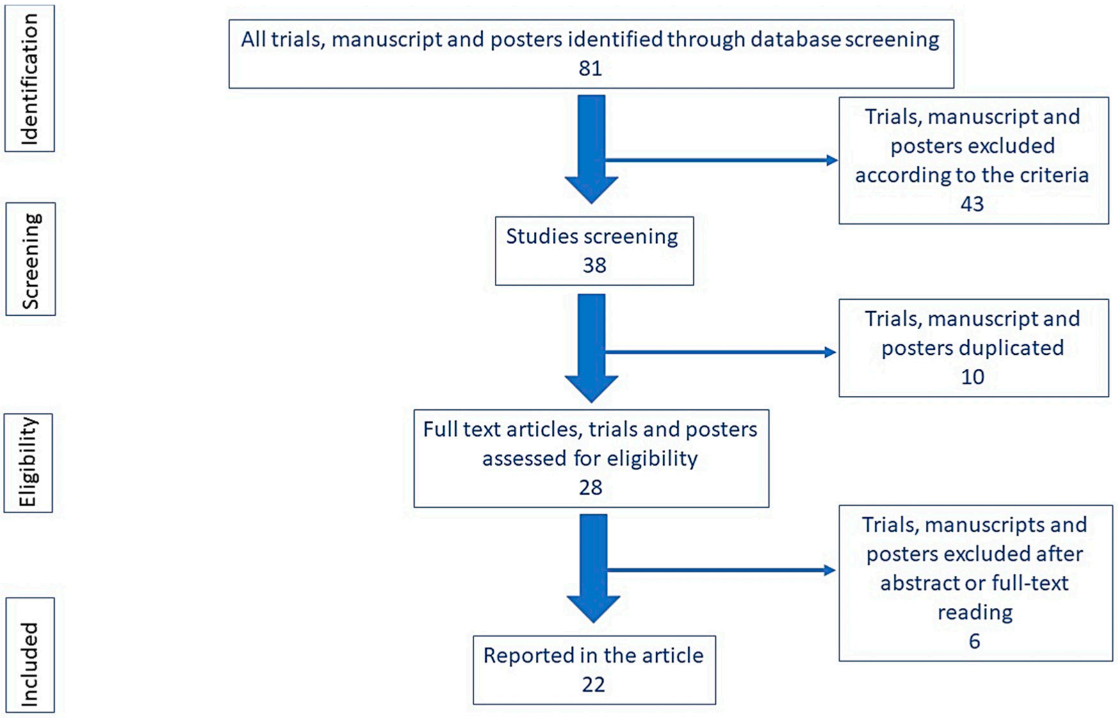

Figure S1. Flow chart of the research strategy. Eighty articles or trials regarding *S. ciferrii complex* human infections were selected by this quantitative research. Forty-two articles were excluded after the application of exclusion criteria. Among the thirty-eight articles or trials eligible for evaluation, ten were excluded as duplicated, while five after abstract or full text reading. Twenty-two articles or trials were considered in this review

Geographic area	America							Europe			Asia					Middle East
	Florida	Georgia	Oregon	Hawaii	Kansas	Brazil	Mexico	France	Austria	Romania	Japan	India	China	Bangladesh	Malaysia	Turkey
N° of isolates	1	1	1	1	1	1	2	18	1	1	1	8	37	12	3	3

Table S1. Geographic distribution of *S. ciferrii* complex human infections. The table shows that Asia is the continent with the highest *S. ciferrii* complex prevalence rate (66%), followed by Europe (22%) and America (9%). In Asia, China is the country with the highest isolation rate (60%); in Europe, France leads with 90% of cases, and in America it is Mexico with 25%.

	**1983**	**1990**	**1995**	**2001**	**2004**	**2011**	**2013**	**2014**	**2015**	**2018**	**2019**	**2021**	**2022**
**N° *S. ciferri* complex isolated from clinical samples**	5	6	12	2	1	1	4	1	7	4	2	33	12

Table S2. Number of *S. ciferrii* complex isolates documented in humans from 1983 to 2022.

	**America**	**Europe**			**Asia**				**Middle East**
	Mexico	France	Austria	Romania	Japan	India	China	Bangladesh	Turkey
Azoles	2	13	1	1	1	2	37	11	1
Echinocandins	0	0	0	0	0	0	0	0	1

Antimetabolites	0	1	0	0	0	3	17	0	0
Polyenes	0	0	0	1	0	0	4	6	1

Table S3. Antifungal resistance in *S. ciferrii* complex strains isolated from humans in different countries. The highest azole-resistance rates have been documented in China, France and Bangladesh. Echinocandin-resistance has been described only in Turkey. China is the country with the highest antimetabolite (5-FU)-resistance rate while resistance to polyenes (AmB) has been described in Bangladesh and China. AmB, Amphotericin B; 5-FU, 5-Fluorocytosine.
